# The efficacy of an extraoral scavenging device on reducing aerosol particles ≤ 5 µm during dental aerosol-generating procedures: an exploratory pilot study in a university setting

**DOI:** 10.1038/s41405-021-00074-5

**Published:** 2021-05-20

**Authors:** Christian Graetz, Paulina Düffert, Ralf Heidenreich, Miriam Seidel, Christof E. Dörfer

**Affiliations:** 1 Clinic of Conservative Dentistry and Periodontology, University Medical Center Schleswig-Holstein, Kiel, Germany; 2 Institute of Air Handling and Refrigeration, Dresden, Germany

**Keywords:** Infection control in dentistry, Dental equipment

## Abstract

**Objective/aim:**

To identify small particle concentrations (eight categories: ≤0.1 µm × ≤5.0 µm) induced by aerosol-generating procedures (AGPs; high-speed tooth preparation, ultrasonic scaling; air polishing) under high-flow suction with a 16-mm intraoral cannula with and without an additional mobile extraoral scavenger (EOS) device during student training.

**Materials and methods:**

Twenty tests were performed (16.94 m^2^ room without ventilation with constant temperature (26.7 (1.1) °C and humidity (56.53 (4.20)%)). Data were collected 2 min before, 2 min during, and 6 min after AGPs. The EOS device and the air sampler for particle counting were placed 0.35 m from the open mouth of a manikin head. The particle number concentration (PN, counts/m3) was measured to calculate ΔPN (ΔPN = [post-PN] − [pre-PN]).

**Results:**

Mean ΔPN (SD) ranged between −8.65E+06 (2.86E+07) counts/m^3^ for 0.15 µm and 6.41E+04 (2.77E+05) counts/m^3^ for 1.0 µm particles. No significant differences were found among the AGP groups (*p* > 0.05) or between the AGP and control groups (*p* > 0.05). With an EOS device, lower ΔPN was detected for smaller particles by high-speed tooth preparation (0.1–0.3 µm; *p* < 0.001).

**Discussion:**

A greater reduction in the number of smaller particles generated by the EOS device was found for high-speed tooth preparation. Low ΔPN by all AGPs demonstrated the efficacy of high-flow suction.

**Conclusions:**

The additional use of an EOS device should be carefully considered when performing treatments, such as high-speed tooth preparation, that generate particularly small particles when more people are present and all other protective options have been exhausted.

## Introduction

Dentistry involves many noninvasive or invasive activities often associated with droplets and aerosols. This is commonly regarded as a potential risk for infections for the whole dental team.^1^ Since the SARS-CoV-2 pandemic, dentistry has been officially classified as one of the very high-risk professions related to disease spread.^2^ Per breath, humans exhale ~10,000–50,000 droplets with a diameter of ~0.5–2 µm. These small particles are able to remain in the air for up to 30 h and can be transmitted by air streams over longer distances.^3,4^ Sneezing increases the size of the droplets tenfold, but as a consequence of their higher weight, these particles sink to the ground within a few seconds. Nevertheless, airborne particles of all sizes can carry potentially pathogenic microorganisms such as viruses and bacteria. Therefore, adequate protective measures against pathogens transmitted via droplets, splatters, or aerosols from patients’ oral cavities are of high importance in dental practices. Studies measuring particle concentrations showed that relatively high concentrations of particles may be present during certain dental procedures.^5,6^ However, this is not completely understood due to the complexity of the interplay between aerosol-generating procedures (AGPs), patients, and dental teams. Particularly, limited data are available for treatments themselves; realistic simulations of mechanical treatment situations have been conducted but the interaction with the breathing patient has not been investigated. Such a situation exists in real life in preclinical dental teaching courses, whereas the treatment situation differs in that situations are simulated by manikin heads to represent the oral cavity.

According to the German national guideline to protect dental staff, many measures are recommended.^2^ This recommendation includes the use of a high-flow suction system combined with proper handling.^2^ When the SARS-CoV-2 pandemic began, the question came up whether the use of additional mobile extraoral scavengers (EOSs) device could be another mitigating factor against airborne particles to provide the highest protection possible for dental staff and patients.^7^ The data of an experimental study^7^ showed that the use of an additional EOS device could further reduce the mean intensity contamination for clinicians and assistants by ~33–76%. Due to their study design, they failed to quantify the contamination risk of smaller particles; however, this quantification seems necessary for more generalized recommendations, as outlined in a recently published German position paper.^8^

Therefore, the current basic in vitro research aimed to identify the possible benefit of an EOS device in addition to an optimally utilized intraoral high-flow suction system to further reduce the particle concentration of aerosols between 0.1 and 5.0 µm in a student training setting in dentistry that is very similar to the clinical situation. These particles were produced by different AGPs, such as (1) tooth preparation with a high-speed rotation handpiece, (2) supra-/subgingival scaling with an ultrasonic scaler, and (3) an air-polishing device (APD) with nonabrasive powder. The hypothesis to be tested was that the reduction in the particle number (PN) concentration during AGPs would be higher with an EOS than without an EOS device.

## Material and methods

### Experimental setup—manikin head and test dental procedure

**Fig. 1:**
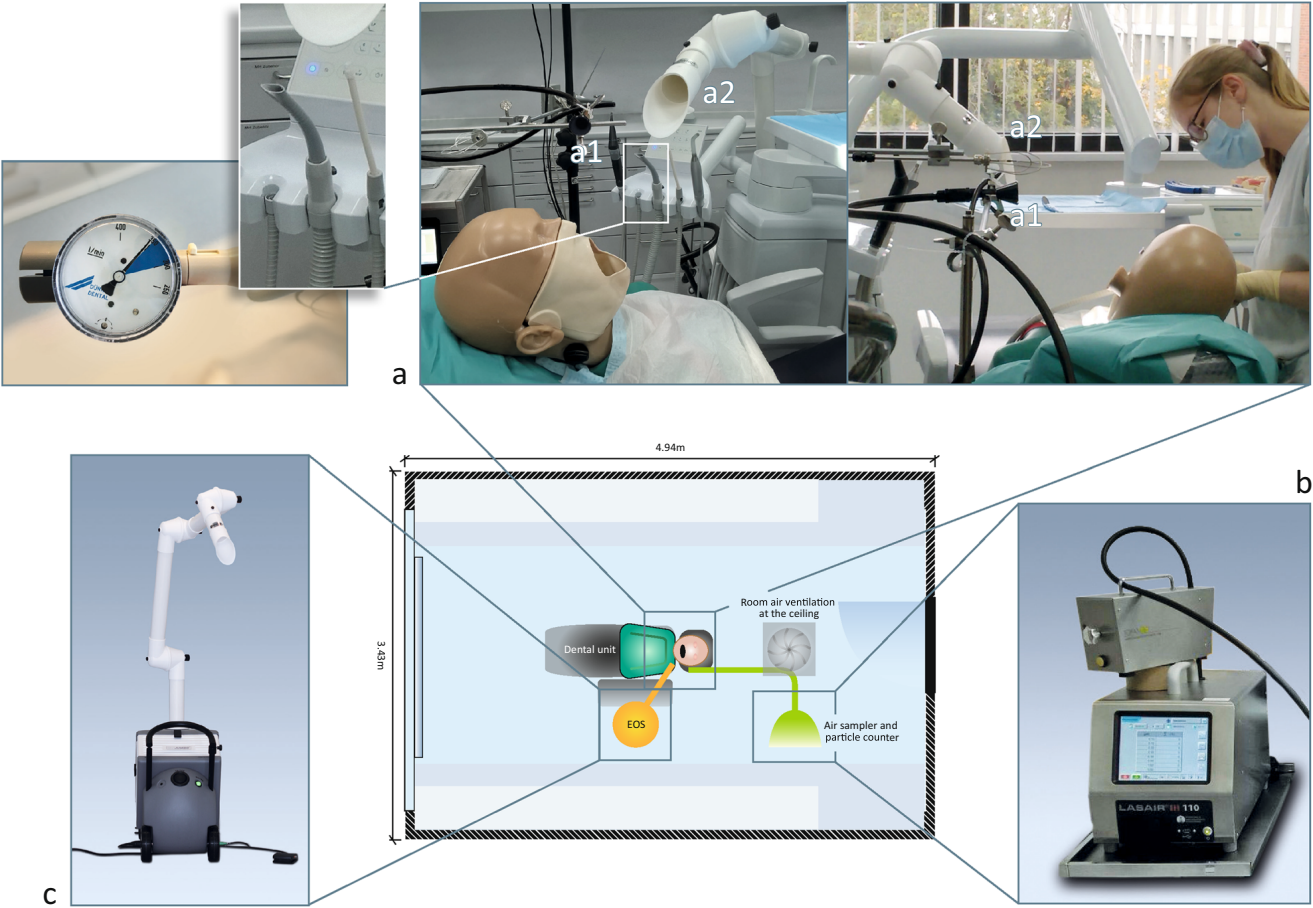
Overview of the experimental setup in the treatment room. **a** Dental unit equipped with a manikin head (Kavo, Biberach, Germany) and a high-flow suction system (Dürr, Bietigheim-Bissingen, Germany) with a 16-mm intraoral cannula and a saliva ejector (left magnification; the calibrated flow rate was ≥350 l/min at the 16-mm intraoral cannula; Dürr, Bietigheim-Bissingen, Germany). a1 Air sample point of the particle counter (Lasair III 110 cleanroom, PMS Inc., USA), sensor for the air velocity and a2 suction tube of the mobile extraoral scavengers (EOS; JakAir Mobile System, ULT, Löbau, Germany) 0.35 m above the mouth of the manikin head. **b** Monitor of the particle counter. **c** View of the EOS device. The EOS device and the air sample point of the particle counter were placed at the same level/distance from the open mouth of the manikin head and the head of the investigator before each test (0.35 m).

To measure the aerosol particles in vitro between 0.1 and 5.0 µm generated by different AGPs, a manikin head (Kavo, Biberach, Germany) was fixed on a dental unit. All tests were performed in a closed room (floor surface 16.94 m^2^, for details please see Fig. 1) without natural ventilation or air conditions at a constant temperature of mean (SD): 26.7 (1.1) °C with an air humidity of ~56.53 (4.20)% in the Clinic of Conservative Dentistry and Periodontology, University Medical Center Schleswig-Holstein, Kiel, Germany, in 2 days in autumn 2020. The study setting simulated student treatment of a manikin head as part of the education in a preclinical study section to avoid unnecessary risks to operators due to the SARS-CoV-2 pandemic situation. Four investigators were inside the test room; two served as students (one treating and one assisting), one served as the supervisor, and one served as the person responsible for the measurement technique. At all times, each investigator wore a surgical mask (3M Deutschland GmbH, Neuss, Germany). The two investigators who served as students wore a face shield (Dental Design oHG, Bad Bramstedt, Germany) over the surgical mask according to internal guidelines for treating noninfective patients during AGPs.

### Aerosol-generating procedures

Each test of an AGP took 10 min in total and was separated into three parts: (1) 2 min during which talking between the operator and patient was simulated as well as other non-AGPs (e.g., local anesthesia) followed by (2) 2 min of AGPs (high-speed tooth preparation and different procedures of professional tooth cleaning), and, last, (3) 6 min of posttreatment activities (e.g., behavior instructions and appointment planning). Different treatment devices were used as AGPs; *n* = 10 tests for high-speed tooth preparation (handpiece at 200,000 rpm; Kavo, Biberach, Germany), *n* = 4 tests for air polishing with nonabrasive powder (APD; LM-Instruments Oy, Pargas, Finland), and *n* = 4 tests for calculus removal with an ultrasonic scaler (US; Kavo, Biberach, Germany). In addition, during two baseline tests (control), no AGP was performed; one test included the use of an EOS device and one the other test did not.

### High-flow suction system and EOSs

During all tests, a high-volume suction cannula with a diameter of 16 mm and a saliva ejector were used in combination with a clinic internal high-flow suction system for reproduceable conditions of flow rate ≥350 l/min (Dürr, Bietigheim-Bissingen, Germany). Prior to each test, the flow rate was calibrated (Fig. 1). The volume of cooling water for high-speed tooth preparation was set to 50 ml/min and was set to 30 ml/min for the ultrasonic scaler; both of these values are in line with the manufacturer’s specifications. The APD was utilized on the lower of two possible levels for the powder stream and on a middle level of water supply (corresponding to ~20–40 ml/min).

To evaluate whether an EOS device might be an additional factor in decreasing or preventing aerosol exposure at dental school, all measurements were performed with and without the use of an EOS device for dentistry (JakAir Mobile System, ULT, Löbau, Germany). Measurement of the PN concentration (counts/m^3^) was always performed during the simulated dental treatment. The EOS device was an additional mobile EOS equipped with a ULPA-U15 filter for removing germs (ULT, Löbau, Germany). It was placed at a 67° angle toward the mouth of the manikin head at a distance of 0.35 m on the left front side and at the same level/distance from the particle counter as the tube end of the air sampler (Lasair III 110 cleanroom, PMS Inc., New Mexico, USA). For details, please see Fig. 1.

### Particle measurements

The concentration of particles with sizes ranging from 0.1 to 5.0 µm was divided into eight categories (0.1, 0.15, 0.2, 0.25, 0.3, 0.5, 1.0, and 5.0 µm) and measured by a particle counter (Lasair III 110 cleanroom, PMS Inc., New Mexico, USA). The logging interval was 20 s, and particle emissions were measured with an air sampler (1.00 CFM flow rate) at a distance of 0.35 m to the open mouth. To keep track of the air conditions in the room, three additional measuring devices were used: single-beam and dual-wavelength nondispersive infrared device (CARBOCAP, Vaisala Oy, Helsinki, Finland) for measuring CO_2_ concentrations; a system for constantly recording the air velocity (Air Velocity Transducer 8475, TSI Inc., Shoreview Minnesota); and a device to measure temperature, air velocity, air pressure, and relative air humidity (TopMessage System, Delphin Technology, Bergisch Gladbach, Germany). During all dental procedures, the air conditioning (AC) system was switched off, and all doors and windows were closed. Between each test, by opening the door and windows as well as by switching on the AC, a comparable base level of temperature and air humidity was re-established.

### Outcomes

Due to the simulation of typical situations with different AGPs in our department in the school of dental medicine, no normal air level for particle evaluation was defined. Therefore, only the difference in PN concentration [ΔPN; post-AGP − pre-AGP, counts/m^3^] for particles with a diameter of 0.1 µm up to 5.0 µm in the eight categories was calculated. The ΔPN was analyzed between tests with/without an additional EOS device and among the three different AGPs.

### Statistical analysis

Data acquisition and collection were performed with Microsoft Excel (Microsoft Excel 16, Microsoft Corporation, One Microsoft Way Redmond, WA, USA). As no sample calculation was performed before investigation, we compared the calculated ΔPN results of all AGPs and the control (without any treatment procedures), then compared the calculated ΔPN results of all AGPs with versus without an EOS device. These calculations were performed separately for each of the particle categories. Therefore, the data sheets were exported to SPSS Statistics (SPSS Statistics 24, IBM, Chicago, IL, USA) for statistical analysis. No normal distribution was detected by Kolmogorov–Smirnov and Shapiro–Wilk tests. Subsequently, a mean value comparison was performed using the Kruskal–Wallis test to detect significant differences according to ΔPN values among the three categories of instruments and the control without AGPs. For the difference between patients with and without the use of an EOS device, the Mann–Whitney *U* test was used. All tests were two-sided; statistical significance was assumed if *p* ≤ 0.05.

## Results

**Fig. 2:**
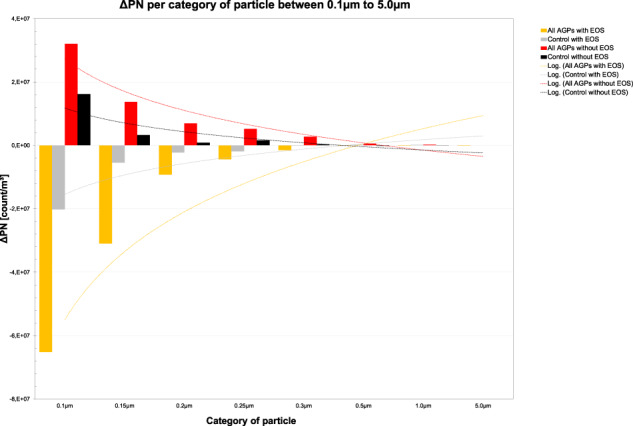
Illustration of the change in particle number concentration (mean (SD) ΔPN: counts/m^3^) for eight categories of particles between 0.1 and 5.0 µm for all aerosol-generating procedures (AGP: high-speed rotation handpiece; air polishing with nonabrasive powder; supra-/subgingival scaling with an ultrasonic scaler) and control tests (no AGP) with and without mobile extraoral scavenger (EOS) device.

We determined an average CO_2_ concentration of 1208 (190) ppm (mean (SD)) during all 20 tests. The air velocity near the air sampling point was 0.07 (0.03) m/s. No statistical significance was identified for any of the room parameters (controlled for CO_2_ concentration, room temperature, air humidity, and air velocity) between the AGP and control groups or between the groups with and without the EOS device (*p* > 0.05). In general, we found decreasing values of ΔPN in the EOS group, whereas we found increasing values in all tests without an EOS device (Fig. 2).

In detail, the difference in ΔPN during dental treatments with an EOS device compared to treatments in which an EOS device was not used was statistically significantly lower for smaller particles with a diameter of 0.1 µm up to 0.3 µm (*p* < 0.001), but not for particles with a diameter of 0.5 µm up to 5.0 µm (*p* = 0.089) (Table 1). In a subgroup analysis, due to the different number of experiments, only the high-speed tooth preparation reached statistical significance for smaller particles with a diameter of 0.1 µm up to 0.3 µm (Table 2, *p* < 0.01).

**Table 1:** Results of the change in particle number concentration (mean(SD) ΔPN: counts/m^3^) for eight categories of particles between 0.1 and 5.0 µm for all aerosol-generating procedures (AGP: high-speed rotation handpiece; air-polishing with nonabrasive powder; supra-/subgingival scaling with an ultrasonic scaler) and control tests (no AGP) with and without mobile extraoral scavengers (EOS) device.

	All AGPs ΔPN [counts/m^3^]	All controls (without AGP) ΔPN [counts/m^3^]	*p* value* between groups of AGP versus control group	All AGPs with EOS ΔPN [counts/m^3^]	All AGPs without EOS ΔPN [counts/m^3^]	*p* value* between groups of AGP with versus without EOS	Control with EOS ΔPN [counts/m^3^]	Control without EOS ΔPN [counts/m^3^]
No. of tests	18	2		9	9		1	1
0.1 µm	−1.66E+07 (5.93E+07)	−2.00E+06 (2.57E+07)	0.853	−6.51E+07 (3.55E+07)	3.20E+07 (3.02E+07)	**<0.001**	−2.02E+07	1.62E+07
0.15 µm	−8.65E+06 (2.86E+07)	−1.10E+06 (6.18E+06)	1.000	−3.10E+07 (1.99E+07)	1.37E+07 (1.51E+07)	**<0.001**	−5.47E+06	3.27E+06
0.2 µm	−1.12E+06 (9.60E+06)	−7.25E+05 (2.23E+06)	0.853	−9.21E+06 (5.00E+06)	6.97E+06 (4.84E+06)	**<0.001**	−2.30E+06	8.50E+05
0.25 µm	4.12E+05 (5.66E+06)	−1.48E+05 (2.43E+06)	1.020	−4.40E+06 (2.23E+06)	5.22E+06 (3.31E+06)	**<0.001**	−1.87E+06	1.57E+06
0.3 µm	6.00E+05 (3.31E+06)	1.33E+05 (2.78E+05)	0.853	−1.52E+06 (2.17E+06)	2.72E+06 (2.91E+06)	**<0.001**	−6.37E+04	3.29E+05
0.5 µm	2.12E+05 (7.44E+05)	−1.59E+04 (2.47E+05)	0.758	−9.30E+04 (3.30E+05)	5.18E+05 (9.26E+05)	0.113	1.59E+05	−1.91E+05
1.0 µm	6.41E+04 (2.77E+05)	6.36E+04 (7.49E+04)	0.442	−6.71E+04 (1.57E+05)	1.95E+05 (3.16E+05)	0.050	1.17E+05	1.06E+04
5.0 µm	−5.88E+02 (4.41E+03)	0	1.000	−1.18E+03 (6.36E+03)	0	0.730	0	0

Bold indicates statistically significant difference.

*Mann–Whitney *U* test was used.

**Table 2:** Subgroup results of the change in particle number concentration (mean(SD) ΔPN: counts/m^3^) for eight categories of particles between 0.1 and 5.0 µm for all aerosol-generating procedures (AGP: high-speed tooth preparation; air-polishing with nonabrasive powder; supra-/subgingival scaling with an ultrasonic scaler) with and without a mobile extraoral scavengers (EOS) device.

Total				
	High-speed tooth preparation ΔPN [counts/m^3^]	Air-polishing with nonabrasive powder ΔPN [counts/m^3^]	Ultrasonic scaling ΔPN [counts/m^3^]	*p* value* between groups of AGP
No. of tests	10	4	4	
0.1 µm	−1.65E+07 (5.47E+07)	−9.82E+06 (9.65E+07)	−2.35E+07 (3.91E+07)	0.968
0.15 µm	−1.07E+07 (2.89E+07)	−2.58E+05 (4.10E+07)	−1.19E+07 (1.84E+07)	0.977
0.2 µm	−1.06E+06 (9.02E+06)	7.35E+05 (1.43E+07)	−3.14E+06 (7.95E+06)	0.956
0.25 µm	3.29E+05 (4.73E+06)	1.48E+06 (9.18E+06)	−4.45E+05 (5.24E+06)	0.994
0.3 µm	4.27E+05 (1.90E+06)	1.81E+06 (6.85E+06)	−1.81E+05 (1.22E+06)	0.859
0.5 µm	1.13E+05 (3.48E+05)	7.23E+05 (1.51E+06)	−5.02E+04 (6.76E+04)	0.581
1.0 µm	3.71E+04 (1.35E+05)	2.04E+05 (5.84E+05)	−7.91E+03 (6.55E+04)	0.698
5.0 µm	7.00E−01 (4.99E+03)	−2.65E+03 (5.30E+03)	0	0.687
Subgroup analyzes without EOS				
	High-speed tooth preparation ΔPN [counts/m^3^]	Air-polishing with nonabrasive powder ΔPN [counts/m^3^]	Ultrasonic scaling ΔPN [counts/m^3^]	*p* value* between groups of AGP
No. of tests	5	2	2	
0.1 µm	2.62E+07 (1.57E+07)	6.89E+07 (4.86E+07)	9.86E+06 (6.58E+06)	0.207
0.15 µm	1.03E+07 (7.60E+06)	3.28E+07 (2.37E+07)	3.05E+06 (2.70E+06)	0.163
0.2 µm	6.08E+06 (3.78E+06)	1.28E+07 (5.82E+06)	3.41E+06 (2.47E+05)	0.19
0.25 µm	4.20E+06 (2.82E+06)	9.21E+06 (3.15E+06)	3.79E+06 (2.18E+06)	0.283
0.3 µm	1.80E+06 (1.76E+06)	6.91E+06 (3.01E+06)	8.26E+05 (3.00E+04)	0.136
0.5 µm	1.99E+05 (4.83E+05)	1.85E+06 (1.12E+06)	2.12E+04 (5.99E+04)	0.136
1.0 µm	7.41E+04 (1.86E+05)	6.51E+05 (3.52E+05)	4.24E+04 (7.07E−01)	0.248
5.0 µm	0	0	0	1.000
Subgroup analyzes with EOS				
	High-speed tooth preparation ΔPN [counts/m^3^]	Air-polishing with nonabrasive powder ΔPN [counts/m^3^]	Ultrasonic scaling ΔPN [counts/m^3^]	*p* value* between groups of AGP
No. of tests	5	2	2	
0.1 µm	−5.91E+07 (4.42E+07)	−8.86E+07 (2.79E+07)	−5.69E+07 (9.71E+06)	0.43
0.15 µm	−3.17E+07 (2.69E+07)	−3.33E+07 (1.06E+07)	−2.69E+07 (1.09E+07)	0.587
0.2 µm	−8.19E+06 (6.44E+06)	−1.13E+07 (1.50E+06)	−9.70E+06 (4.20E+06)	0.761
0.25 µm	−3.54E+06 (2.20E+06)	−6.26E+06 (1.92E+06)	−4.68E+06 (2.47E+06)	0.266
0.3 µm	−9.49E+05 (5.61E+05)	−3.28E+06 (5.30E+06)	−1.19E+06 (6.57E+05)	0.608
0.5 µm	2.76E+04 (1.46E+05)	−4.08E+05 (7.12E+05)	−7.92E+04 (8.20E+04)	0.464
1.0 µm	2.60E+00 (5.08E+04)	−2.44E+05 (3.15E+05)	−5.82E+04 (5.23E+04)	0.238
5.0 µm	1.40E+00 (7.49E+03)	−5.30E+03 (7.49E+03)	0	0.648
	*p* value** between test with EOS versus without EOS	*p* value** between test with EOS versus without EOS	*p* value** between test with EOS versus without EOS	
0.1 µm	**0.007**	0.333	0.333	
0.15 µm	**0.007**	0.333	0.333	
0.2 µm	**0.008**	0.333	0.333	
0.25 µm	**0.008**	0.333	0.333	
0.3 µm	**0.007**	0.333	0.333	
0.5 µm	0.548	0.333	0.667	
1.0 µm	0.690	0.333	0.333	
5.0 µm	1.000	0.667	1.000	

*Kruskal–Wallis test.

**Mann–Whitney *U* test was used.

An overview of the subgroup analysis is given in Table 2. Independent of using an additional EOS device, neither significant differences among the ΔPN values of all particle categories induced by the three different AGP devices (*p* > 0.05) nor between the AGP versus control groups (*p* > 0.05) were detectable.

Furthermore, no significant ΔPN difference was determined among the test days (*p* > 0.05).

## Discussion

It is indisputable that airborne particles carry a risk of disease transmission. To protect patients and dental staff, the particle concentration during dental procedures needs to be minimized. As SARS-CoV-2 is transmitted via airborne particles, aerosol exposure during dental procedures and its reduction have gained worldwide interest as an effective approach to protect dental staff and patients.^2^

Our data showed no relevant differences between AGPs and the control or among the different AGPs when a high-flow suction system was used. For all AGPs, the additional use of a mobile EOS device led to a significantly lower concentration of particles between 0.1 and 0.3 µm in diameter (Table 1, *p* *<* 0.001). Evaluating the AGPs separately showed a similar difference with and without EOS devices in all groups; however, due to the different number of experiments, only the high-speed tooth preparation reached statistical significance (Table 2, *p* < 0.01). The measured efficacy of particle reduction with extraoral scavenging devices is in accordance with another study with a similar focus on safety in dentistry.^7^ However, the results of experimental studies have to be interpreted with caution, as they cannot be directly extrapolated to a clinical situation with aerosol generation by a patient who breathes, potentially sneezes, or coughs and cannot be extrapolated to clinical situation regarding the risk of infection. It must be assumed that with a decrease in human aerosols, the total number of potentially infectious particles will also decrease and lead to a lowered risk of aerogenic infection with or without AGPs.

The standardization of our experimental setup with a manikin instead of a patient and an additional supervisor in the room, therefore, delivers reliable and reproducible data on the one hand but, on the other hand, limits the credibility of an extrapolation to clinical situations. However, the setting used in this study, as it is also used in preclinical dental education, simulates the clinical situation in detail with artificial cheeks, a realistic mouth opening, and the same AGPs and suction devices, especially in treatment situations, where a rubber dam is used as a barrier against potentially infectious biological materials. It may be suspected that the differences are neglectable. Although the generated aerosol particles were measured precisely, we are far from simulating the complete complexity of a living patient in such a situation in general. Explicitly, this is true for treatment sessions, such as diagnostic steps, low-speed caries excavation, scaling manually, or localized application of antiseptics, that do not require the use of a high-flow suction system.

Another limitation of our experimental setup is that we only controlled the room temperature and humidity conditions during a period of ~10–15 min between each test run but did not calibrate the PN baseline for practical reasons. To compensate for that, ΔPN instead of absolute PN values were analyzed. However, during that time span, conventional airing through windows, doors, and AC with a high percentage of fresh air for 15 min has been shown to effectively reduce the particle concentration in the room.^9^

In contrast to the results of a comparable experimental study on dental manikins with a high-volume suction system (bore diameter of 8 mm),^7^ we found that larger particles with a size of 0.5 µm up to 5 µm showed no significant difference in ΔPN among all AGPs with versus without the addition of a mobile EOS device. We can hypothesize that this result is due to another type of dental suction system (high-flow, bore diameter 16 mm) used in our study, which was so powerful that particles with a size ≥ 0.5 µm were eliminated immediately,^10^ whereas smaller particles were less affected by suction due to their smaller surface area and remained in the air. It could be assumed that such small particles would be sampled by the EOS device as its opening was directly above the manikin head and the air vent of our particle sampler was diagonally above the manikin head (Fig. 1). Therefore, in cases with a large amount of aerosolized particles, e.g., high-speed tooth preparation with a large amount of kinetic energy through the rotation of the bur,^11^ an additional EOS device may contribute to a quantitative reduction in small droplet aerosols.

Moreover, we assumed that larger droplets and particles would be immediately reduced near the mouth opening or may be eliminated by the high-flow suction system with a 16-mm intraoral cannula.^10,12^ A recent meta-analysis with four RCTs found similar effects for high-volume evacuators, which reduced contamination in aerosols near the patient’s mouth (~0.3 m) but not at longer distances.^13^ Surprisingly, one split-mouth RCT found no significant difference in the efficacy of reducing aerosols by the use of a high-volume evacuator compared to a conventional dental suction with a saliva ejector or a low-volume evacuator at 0.4 or 1.5 m with ultrasonic scaling as the AGP.^14^

It seems well known that to reduce the risk of aerosols in dentistry, more than one protective measure has to be applied.^2,15^ A recently published systematic review^16^ recommended combining strategies of protective procedures, including preprocedural antimicrobial oral rinsing and the use of a high-volume evacuator with a properly sized suction cannula and rubber dam. However, more options than these three measures are mentioned in the scientific literature, e.g., four-handed dentistry, room ventilation, masks, and frequent disinfection of the suction system of the dental unit.^2,15,17^ Whenever some of these protective procedures are not possible or their use is limited, especially ventilation of the room,^8^ a mobile EOS with a HEPA filter (high-efficiency particulate air) could help to reduce the particle concentration further and faster, as indicated by our current data. We calculated an air exchange rate of 3 h^−1^ for the tested mobile EOS device. This is in line with other similar investigations in dentistry.^7,18^ However, according to the statement of the German Society of Hospital Hygiene (DGKH), the recommended rate should be at least 2 h^−1^ and will be beneficial at 5 h^−1^ to provide a safer working environment.^19^ In addition, it should be noted that EOS devices work according to the recirculation principle, which also means that even in continuous operation, only a fraction of the room air is cleaned. With a high turnover rate, generously dimensioned units are necessary, especially in large rooms with many workplaces, such as rooms with manikin heads, in the context of student training.

It must be said that the acquisition and maintenance costs of an EOS device are quite high, as they require professional installation and regular changes of the potentially contaminated filters; also, their use creates noise, which could lead to discomfort.^8,20^ Moreover, we cannot conclude from our data that EOS devices are more effective than, for example, opening a window for 15 min between treatments.^9^ Opening a window is an easy, cheap, and yet effective way to reduce floating airborne particles with air ventilation,^2,3^ even though the effectiveness of ventilation through open windows varies widely depending on the weather and other factors. We suggest that before routine use of EOS devices in dentistry, professional staff should be more aware of health risks, working habits, and economic factors.^21^

Furthermore, no difference could be detected among the various AGPs and the control for particle sizes between 0.1 and 5.0 µm. This is contrary to other investigations. For instance, a recently published experimental study of air sampling on the backside of the manikin head with a spectrometer showed that the instrument type (high-speed rotation handpiece and ultrasonic scaler) and spray direction significantly influenced the resulting aerosol concentration only for smaller particles <1 µm on the backside of the manikin head.^9^ They found that aerosol generation by an ultrasonic scaler is lower than that by a high-speed turbine. Moreover, the control efficiency might depend on exactly how the instrument is used during a treatment.^9^ Similar to our study results, Nulty et al.^22^ showed by means of a particle counter in particle size categories of 1–10 µm that smaller sized particles (≤1 µm) generated by various AGPs appeared to remain within the same range sampled as the control measurements with no procedure taking place. Although unlikely, it cannot be excluded that this was due to the experimental setting with treatments conducted on a manikin head.^9–11^ It could also be assumed that according to the outlined methods for particle sampling, the particle concentration generated by AGPs is superimposed by background particle movements and the differences in equipment used for measurements.^5^ Therefore, we analyzed ΔPN to reduce the possible impact of people moving inside the room as one factor that could result in a higher air turbulence with higher resuspension of particle deposition on surfaces and the ground inside the room.^23^ To be able to detect errors from such effects, we monitored the air velocity near the point of air sampling (air samplers using a small air vent for particle sampling). We found a low average air velocity without a significant difference between all tests (*p* = 0.485) and concluded that all variations were due to natural thermal effects. The relatively high CO_2_ concentration in our investigation is worth mentioning, as it may indicate a high amount of exhaled air in the room. One could argue that according to the high amount of exhaled air in the room, all four investigators could have been an additional particle source, as CO_2_ and particles are exhaled together.^24^ However, the decisive factor here is that every investigator in the room wore a certified surgical mask according to the EN 14683 standard at all times, providing a barrier against particles but not CO_2_ gas.

Finally, we have to critically discuss the relatively short duration of 10 min of data collection, with only 2 min used for the AGP procedure. It should be kept in mind that this is a very short exposure duration, and dental AGP procedures would probably take more time. This may also have partially resulted in the low contamination levels identified. For smaller sized particles of 1 µm or less, Kun-Szabo et al.^9^ identified a ΔPN of –7.0E+05 counts/m^3^ up to 8.0E+03 counts/m^3^ for nearly the triple time duration of AGPs (5.4 min) by utilizing a high-volume evacuator system. The lower value of particle reduction is similar to our ΔPN of −7.25E+05 counts/m^3^, whereat the upper value is higher at 6.4E+04 counts/m^3^, perhaps a consequence of the shorter time duration for AGP or the higher suction volume of the high-flow suction system used in our setting.

Although these limitations and restrictions of an experimental study should be taken into account when trying to draw conclusions for “real” clinical dental treatment, the current findings help to improve the present understanding of the effects of different therapeutic and protective procedures and provide highly reliable and reproducible data. Nevertheless, clinical studies are needed to analyze the risk of airborne infections during dental treatment in its full complexity.

## Conclusions

Within the limits of the present experimental pilot study to simulate students’ training setting, a high-flow suction system with a 16-mm intraoral cannula significantly reduced the number of generated particles during different AGPs. The beneficial effect of aerosol reduction of a mobile extraoral scavenging device in a simulated education situation with more people inside a room could be considered when all other protective options have already been exhausted and AGPs, such as high-speed tooth preparation, generate particularly small particles (0.1–0.3 µm).
